# DCE-MRI is more sensitive than IVIM-DWI for assessing anti-angiogenic treatment-induced changes in colorectal liver metastases

**DOI:** 10.1186/s40644-021-00436-0

**Published:** 2021-12-19

**Authors:** Mihaela Rata, Khurum Khan, David J Collins, Dow-Mu Koh, Nina Tunariu, Maria Antonietta Bali, James d’Arcy, Jessica M Winfield, Simona Picchia, Nicola Valeri, Ian Chau, David Cunningham, Matteo Fassan, Martin O Leach, Matthew R Orton

**Affiliations:** 1 Department of Radiology, MRI Unit, The Royal Marsden NHS Foundation Trust, London, United Kingdom; 2 Division of Radiotherapy and Imaging, The Institute of Cancer Research, London, United Kingdom; 3 Department of Medicine, GI and Lymphoma Unit, The Royal Marsden NHS Foundation Trust, London and Sutton, United Kingdom; 4 Cancer Research UK National Cancer Imaging Translational Accelerator (NCITA), London, United Kingdom; 5 Centre for Evolution and Cancer, The Institute of Cancer Research, London and Sutton, United Kingdom; 6 Division of Surgery and Cancer, Faculty of Medicine, Imperial College London, London, United Kingdom; 7 Department of Medicine (DIMED), Surgical Pathology Unit, University of Padua, Padua, Italy; 8 Veneto Institute of Oncology IOV-IRCCS, Padua, Italy; 9 Royal Marsden NHS Foundation Trust & Institute of Cancer Research, Downs Road, SM2 5PT Sutton, London, UK

**Keywords:** Dynamic contrast enhanced MRI (DCE-MRI), Intravoxel incoherent motion diffusion weighted imaging (IVIM-DWI), Perfusion, Colorectal liver metastasis, Clinical trial.

## Abstract

**Background:**

Diffusion weighted imaging (DWI) with intravoxel incoherent motion (IVIM) modelling can inform on tissue perfusion without exogenous contrast administration. Dynamic-contrast-enhanced (DCE) MRI can also characterise tissue perfusion, but requires a bolus injection of a Gadolinium-based contrast agent.

This study compares the use of DCE-MRI and IVIM-DWI methods in assessing response to anti-angiogenic treatment in patients with colorectal liver metastases in a cohort with confirmed treatment response.

**Methods:**

This prospective imaging study enrolled 25 participants with colorectal liver metastases to receive Regorafenib treatment. A target metastasis > 2 cm in each patient was imaged before and at 15 days after treatment on a 1.5T MR scanner using slice-matched IVIM-DWI and DCE-MRI protocols.

MRI data were motion-corrected and tumour volumes of interest drawn on b=900 s/mm^2^ diffusion-weighted images were transferred to DCE-MRI data for further analysis. The median value of four IVIM-DWI parameters [diffusion coefficient D (10^−3^ mm^2^/s), perfusion fraction f (ml/ml), pseudodiffusion coefficient D* (10^−3^ mm^2^/s), and their product fD* (mm^2^/s)] and three DCE-MRI parameters [volume transfer constant K^trans^ (min^−1^), enhancement fraction EF (%), and their product KEF (min^−1^)] were recorded at each visit, before and after treatment.

Changes in pre- and post-treatment measurements of all MR parameters were assessed using Wilcoxon signed-rank tests (*P*<0.05 was considered significant). DCE-MRI and IVIM-DWI parameter correlations were evaluated with Spearman rank tests.

Functional MR parameters were also compared against Response Evaluation Criteria In Solid Tumours v.1.1 (RECIST) evaluations.

**Results:**

Significant treatment-induced reductions of DCE-MRI parameters across the cohort were observed for EF (91.2 to 50.8%, *P*<0.001), KEF (0.095 to 0.045 min^−1^, *P*<0.001) and K^trans^ (0.109 to 0.078 min^−1^, *P*=0.002). For IVIM-DWI, only D (a non-perfusion parameter) increased significantly post treatment (0.83 to 0.97 × 10^−3^ mm^2^/s, *P*<0.001), while perfusion-related parameters showed no change. No strong correlations were found between DCE-MRI and IVIM-DWI parameters. A moderate correlation was found, after treatment, between K^trans^ and D* (*r*=0.60; *P*=0.002) and fD* (*r*=0.67; *P*<0.001). When compared to RECIST v.1.1 evaluations, KEF and D correctly identified most clinical responders, whilst non-responders were incorrectly identified.

**Conclusion:**

IVIM-DWI perfusion-related parameters showed limited sensitivity to the anti-angiogenic effects of Regorafenib treatment in colorectal liver metastases and showed low correlation with DCE-MRI parameters, despite profound and significant post-treatment reductions in DCE-MRI measurements.

**Trial registration:**

NCT03010722 clinicaltrials.gov; registration date 6^th^ January 2015.

**Supplementary Information:**

The online version contains supplementary material available at 10.1186/s40644-021-00436-0.

## Background

Regorafenib (Stivarga®), a small molecule multiple kinase inhibitor with action against pro-angiogenic and pro-proliferative targets, has been shown to prolong disease survival for colorectal cancer patients [1]. However, treatment using Regorafenib is associated with side effects in up to 98% of patients [2]. Hence, early identification of patients who are benefiting from treatment can help to individualize treatment, by potentially terminating ineffective treatments and minimizing drug toxicity.

Functional MRI techniques, such as dynamic contrast enhanced (DCE)-MRI and diffusion weighted imaging (DWI), provide information about perfusion and cellularity. DCE-MRI can characterise tissue vascularisation [3, 4], but requires repeated rapid image acquisition of the tumour, sustained over a few minutes after bolus injection of a Gadolinium-based contrast agent. The temporal evolution of enhancement on T1-weighted imaging is used to model and derive quantitative vascular parameters. Studies have shown that DCE-MRI parameters can provide insights into early therapeutic effects or disease outcome [5]. In colorectal liver metastases treated with Regorafenib, an early reduction in KEF (product of transfer constant K^trans^ and enhancing fraction EF) value by >70% was associated with better disease control [6].

Diffusion-weighted imaging provides insights into tissue water mobility and does not require exogenous contrast injection. By performing DWI using multiple b-values combined with intravoxel incoherent motion (IVIM) modelling, tissue water diffusion is expected to be separated from pseudo-diffusion that can reflect tissue perfusion [7, 8]. IVIM-DWI perfusion measurements can help characterization and assess response to anticancer treatments [9]. However, clinical IVIM-DWI studies [10–12] in colorectal liver metastases have been inconclusive about the value of the perfusion-sensitive IVIM parameters for assessing drug therapeutic effects. This may be related to the fact that colorectal liver metastases are predominantly hypovascular and their IVIM-DWI derived parameters have poor measurement repeatability [13], which can limit the sensitivity of the technique in detecting vascular changes.

Previous studies compared measurements of vascular parameters using DCE-MRI with IVIM-DWI in rectal cancer [14, 15]. A similar assessment of the two techniques in colorectal liver metastases will aid understanding the relative merits of both techniques in their deployment in clinical practice. Hence, the aim of this study is to compare the use of DCE-MRI and IVIM-DWI techniques in assessing the response to Regorafenib treatment in colorectal liver metastases. Note that this work follows up a clinical report of the efficacy of drug treatment with Regorafenib [6]. In the present study, we compare IVIM-DWI measurements (previously not reported) against DCE-MRI measurements.

## Methods

### Study population

The trial recruitment was approved by our institutional review board (National Research Ethics Service, NRES Committee London-Fulham; Research Ethics Committee reference 14/LO/1812) and informed written consent was obtained from each patient.

**Fig. 1:**
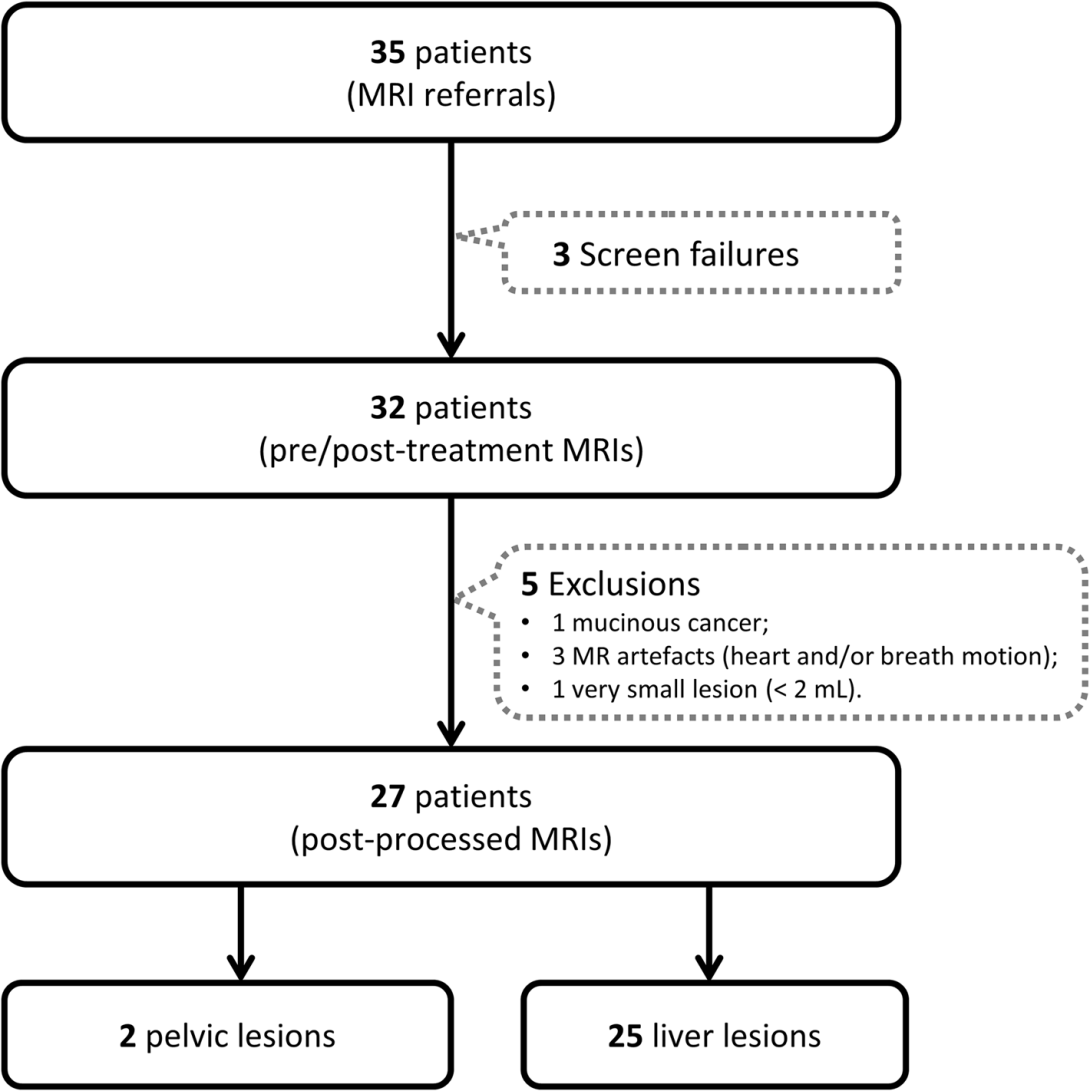
Flowchart of the MR cohort

This prospective phase II study (NCT03010722 registry number on clinicaltrials.gov) enrolled 25 patients with colorectal liver metastases, between March 2015 and May 2016, to a single drug Regorafenib treatment. The three inclusion criteria were: (1) patients at least 18y-old with a WHO performance status of 0-1 for which all conventional treatments were exhausted; (2) patients had metastatic disease amenable to biopsy and repeat measurements with DCE-MRI; (3) patients were confirmed to have a RAS-mutant cancer type. The patient cohort, including case exclusions, is presented in Fig. 1.

In each patient, a target metastasis (>2 cm) was selected to undergo DCE-MRI and IVIM-DWI before (day -7 to 0) and at day 15 after treatment. Five additional patients with liver metastases were recruited (February to June 2017) to assess same-day test-retest repeatability of the IVIM-DWI technique used in the prospective study.

### MR Image acquisition

All imaging was performed on a 1.5T MR scanner (MAGNETOM Avanto, Siemens Healthcare, Erlangen, Germany), see full parameters in Table 1. The IVIM-DWI scans were performed prior to DCE-MRI studies to avoid possible effects of gadolinium contrast on the IVIM-DWI measurements.

**Table 1:** MR parameters for the IVIM-DWI and DCE-MRI sequences.

MRI Parameters	Liver tumours (*n*=25 patients)	
	**IVIM-DWI**	**DCE-MRI**
**Sequence**	**2D single shot echo planar imaging**	**3D gradient echo**
**Acquisition plane**	Coronal	Coronal
**Breathing mode**	Free breathing	Breath holding
**Total acquisition time [min:s]**	10:25	04:18
**Time per single acquisition [min:s]**	02:05	04:18
**Number of averages**	5	1
**Acquired voxel size [mm**^**3**^**]**	3.1 × 3.1 × 5	3.1 × 3.1 × 5
**Reconstructed voxel size [mm**^**3**^**]**	1.56 × 1.56 × 5	1.56 × 1.56 × 5
**Slice thickness [mm]**	5	5
**TR [ms]**	5000	3
**TE [ms]**	60	0.89
**Flip angle [˚]**	-	11
**Slices per slab**	20	14
**Slice gap [mm]**	0	0
**Slice oversampling [%]**	-	14.3
**Matrix (FE x PE)**	128 × 128	128 × 128
**FOV [mm**^**2**^**]**	400 × 400	400 × 400
**Dynamic measurements**	-	40
**Breath holding** (pause 6 s after dyn2, dyn4,etc.)	no	yes
**Receiver bandwidth [Hz/Pixel]**	1860	650
**Parallel acquisition (GRAPPA) (PE acceleration factor x reference lines)**	2 × 30	2 × 24
**Phase partial Fourier**	7/8	no
**Slice partial Fourier**	-	6/8
**Fat suppression**	SPAIR	none
**8 b-values [s/mm**^**2**^**]**	0, 20, 40, 60, 120, 240, 480, 900	-
**Diffusion times [ms]**	δ = 14.6; Δ = 24	-

*TR=repetition time; TE=echo time*

*FE=frequency encoding; PE=phase encoding; FOV=field of view*

*GRAPPA=GeneRalized Autocalibrating Partial Parallel Acquisition*

*SPAIR=SPectral Attenuated Inversion Recovery*

IVIM-DWI protocol. Free-breathing coronal IVIM-DWI was acquired using a 2D echo planar imaging sequence with a 3 direction scan trace method and 8 b-values [range 0-900 s/mm^2^]. DWI for each gradient direction was obtained and 5 independent acquisitions (no averaging) were acquired over 10 min.

DCE-MRI protocol. A standard dose of contrast agent (Dotarem, 0.2 ml/kg) followed by 20 ml of saline were delivered by an automatic power injector at 3 ml/s. Breath-hold coronal DCE-MRI data were acquired using a 3D spoiled gradient echo sequence (VIBE-volumetric interpolated breath-hold examination) that matched the field of view and resolution of IVIM-DWI. Dynamic scans were preceded by a calibration scan with the same parameters, but at a lower flip angle (2°) and with 7 averages, to enable contrast quantification [16]. Patients were imaged using a sequential breath-hold technique optimised for liver lesions [17]: two imaging volumes were acquired during each 6 s breath-hold, followed by a 6 s breathing gap; a total of 40 volumes were acquired over 4 min.

### MRI data processing

**Fig. 2:**
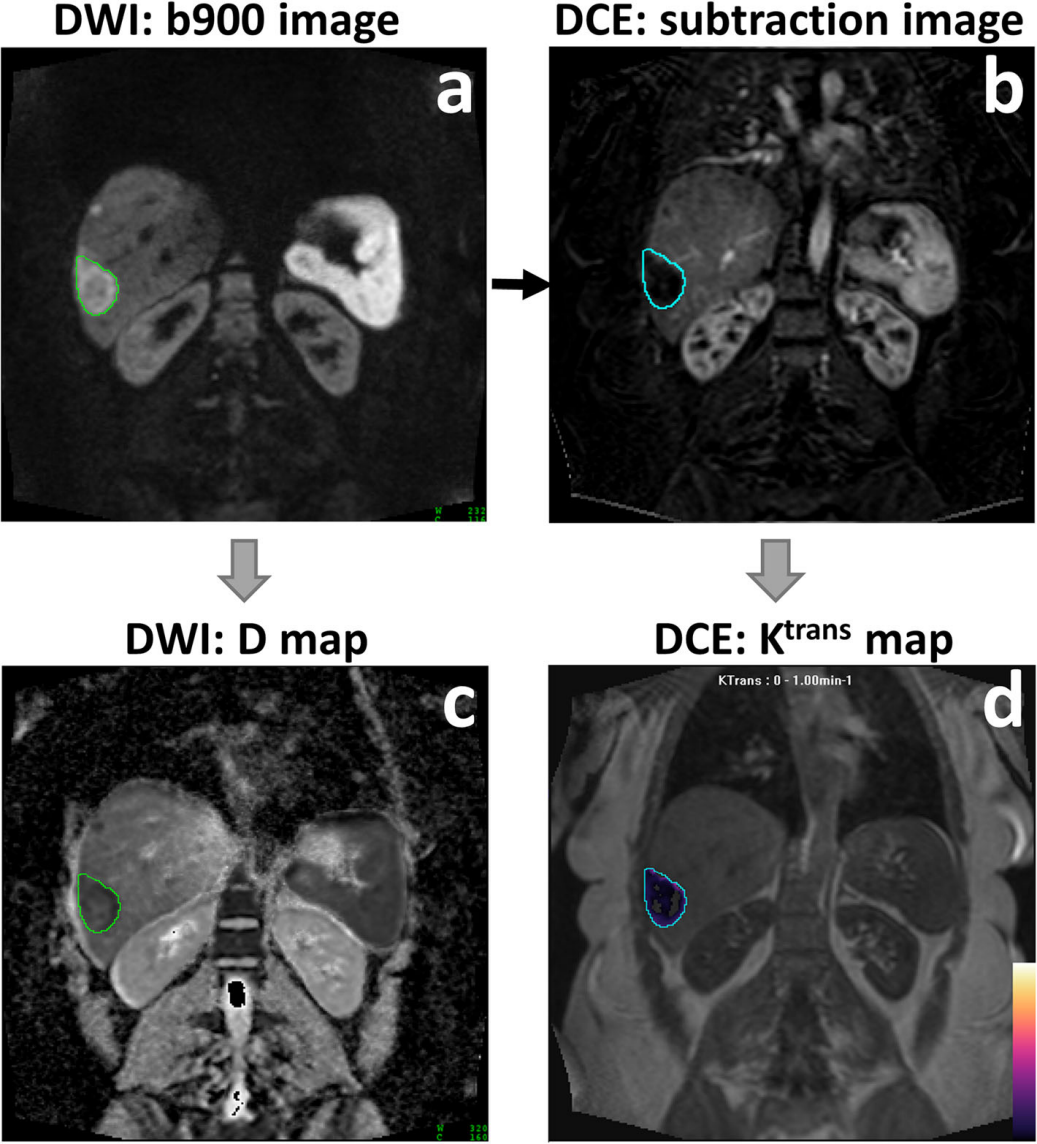
VOI-based data processing (example of one slice out of the 8 evaluated) for a 48 year old female patient with a lesion in segments 7/8 of the liver. VOI was drawn on the highest-b-value image (**a**), then transferred to the DCE-MRI subtraction image (**b**). The subtraction image was calculated as the difference between the dynamic image with peak enhancement within the liver parenchyma (dynamic 9/40) and the first pre-contrast image (dynamic 1/40). The VOIs were used in conjunction with each of the computed maps to derive the median values of parameters of interest: D (**c**) and K^trans^ (**d**) are shown here. Note that K^trans^ map is shown overlaid on the last dynamic image of the DCE-MRI acquisition (dynamic 40/40)

MRI data were motion-corrected (2D techniques, Matlab 2017a, see Supplementary material), and tumour volumes of interest (VOI) were drawn by an experienced radiologist on high-b-value IVIM-DW images (b900) for analysis, see Fig. 2. The VOIs were transferred to the DCE-MR images for quantitative analysis. Voxel-wise analysis of the delineated VOI was performed using an in-house software designed for each imaging technique, and median values of the parameters of interest for both IVIM-DWI and DCE-MRI were reported before and after treatment for every patient.

IVIM-DWI data. A model using a Markov random field approach generated IVIM-DWI estimates using a Matlab script (Matlab 2017a; see Supplementary material). The four IVIM-DWI parameters were: diffusion coefficient D, perfusion fraction f (proportion of a voxel volume occupied by capillaries), pseudo-diffusion coefficient D* (convective motion of blood in vessel network), and their product fD*.

DCE-MRI data. The pharmacokinetic analysis used the extended Kety/Tofts model [18, 19] in conjunction with a cosine-based arterial input function model [20] derived from population-averaged values [21] and was applied using a software from [22].The following main DCE-MRI parameters were reported: volume transfer constant between plasma and extracellular extravascular space (K^trans^) and the enhancement fraction (EF). The EF was defined as percentage of voxels within the VOI that enhance above the noise floor. A voxel was considered to be enhanced if its post-contrast signal intensity was at least one standard deviation above the mean pre-contrast signal for a period of 60 s after the arrival of contrast in the lesion.

Parameters were computed to account for additional necrosis after treatment, which typically manifests by non-enhancement. Two tumour-derived K^trans^ estimates were reported: the whole tumour K^trans^(all), and valid voxels only K^trans^(nonzeros), i.e. excluding all non-enhancing voxels. Potential change in the volume of enhancing tumour after treatment (such as new necrosis) was accounted for by reporting a new parameter which incorporates both effects: KEF= K^trans^(nonzeros) x EF [23]. Note that this parameter is the product of the summarized median values of its two components, and does not rely on a direct voxel-wise approach.

### Statistical analysis

Treatment-induced changes on all parameters were assessed with Wilcoxon signed-rank tests as parameters were not normally distributed. Correlations between DCE-MRI (K^trans^, KEF) and IVIM-DWI (f, D*, fD*) parameters were assessed by Spearman rank tests. The IVIM-DWI repeatability was assessed with Bland-Altman analysis. For all analyses, a P-value of <0.05 was deemed statistically significant.

## Results

### Patient demographics

All 25 study patients (16 men, 9 women; mean age 64.4 ±10.7 years) successfully completed all imaging studies and their clinical characteristics are presented in Table 2. Another 5 patients (3 men, 2 women; mean age 61.0 ±11.6 years) underwent short term test-retest repeatability studies for the IVIM-DWI method only (Table 2).

**Table 2:** Clinical characteristics of patients for the two cohorts

Main cohort (*N*=25): phase II clinical trial patients; DCE-MRI vs. IVIM-DWI	
Disease	liver metastases from colorectal cancer
Primary cancer	colorectal (all patients)
Treatment	oral anti-angiogenic drug (Regorafenib) administered daily
Sex [Female/Male]	9/16
Age in years (mean ± SD; range)	64.4 ± 10.7; 44 - 86
Lesion volume in mL (mean ± SD; range)	45.8 ± 60.1; 2.2 - 265.1
**Repeatability cohort** **(*N*=5): clinical patients; IVIM-DWI**	
Disease	liver metastases from gastrointestinal cancer
Primary cancer	3 colorectal, 1 stomach, 1 caecal
Treatment	not relevant
Sex [Female/Male]	2/3
Age in years (mean ± SD, range)	61.0 ± 11.6; 50 - 80
Lesion volume in mL (mean ± SD, range)	91.9 ± 164.5; 4.1 - 385.6

*SD=standard deviation*

### DCE-MRI and IVIM-DWI assessment of treatment response

**Fig. 3:**
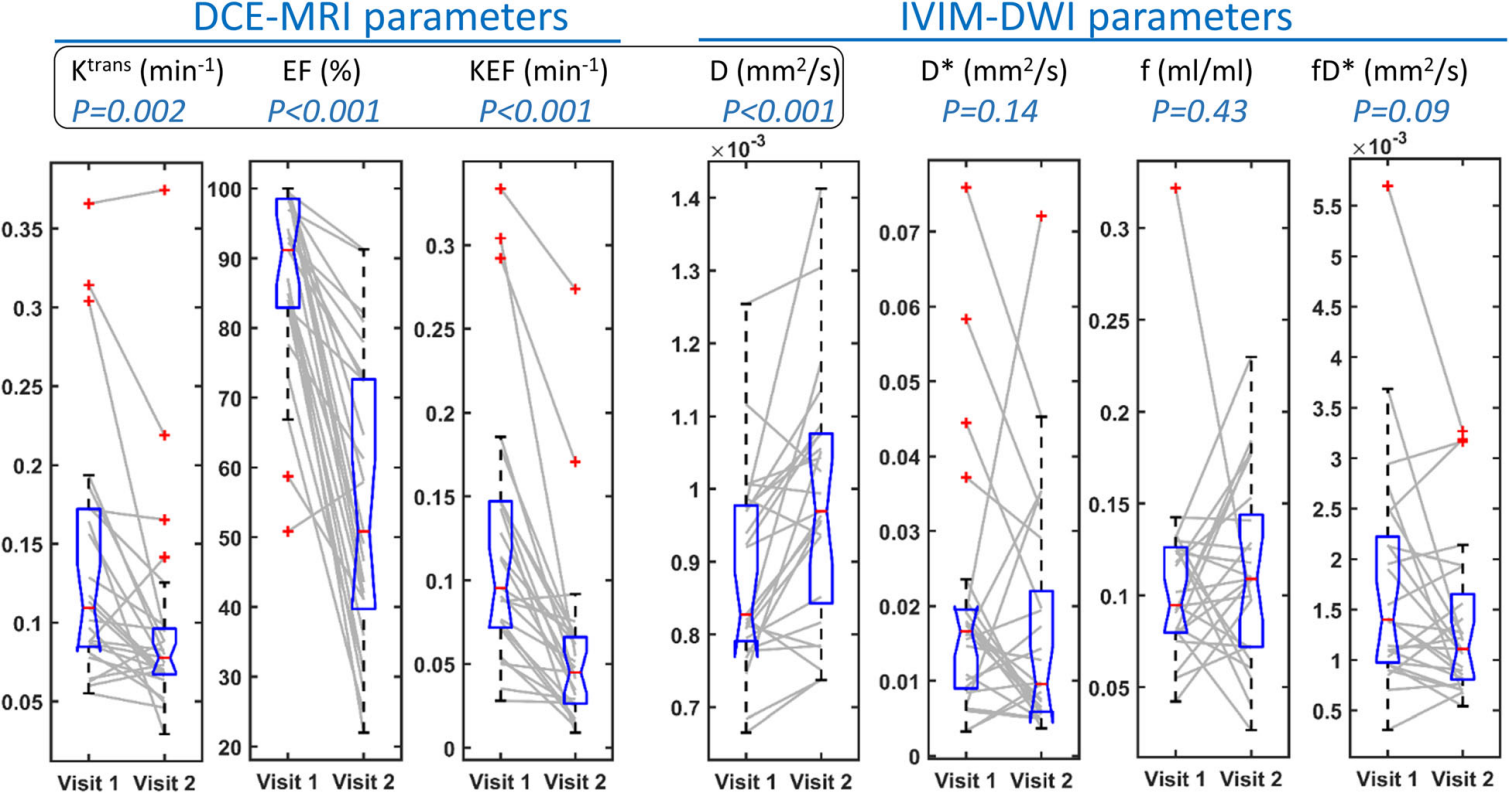
Pre/post-treatment overlapped Ladder/Box plots of MR parameters from 25 liver metastases patients demonstrating significant response for all DCE-MRI parameters and D, but no significance for the three other IVIM-DWI parameters. Wilcoxon P-values are listed within the header

**Fig. 4:**
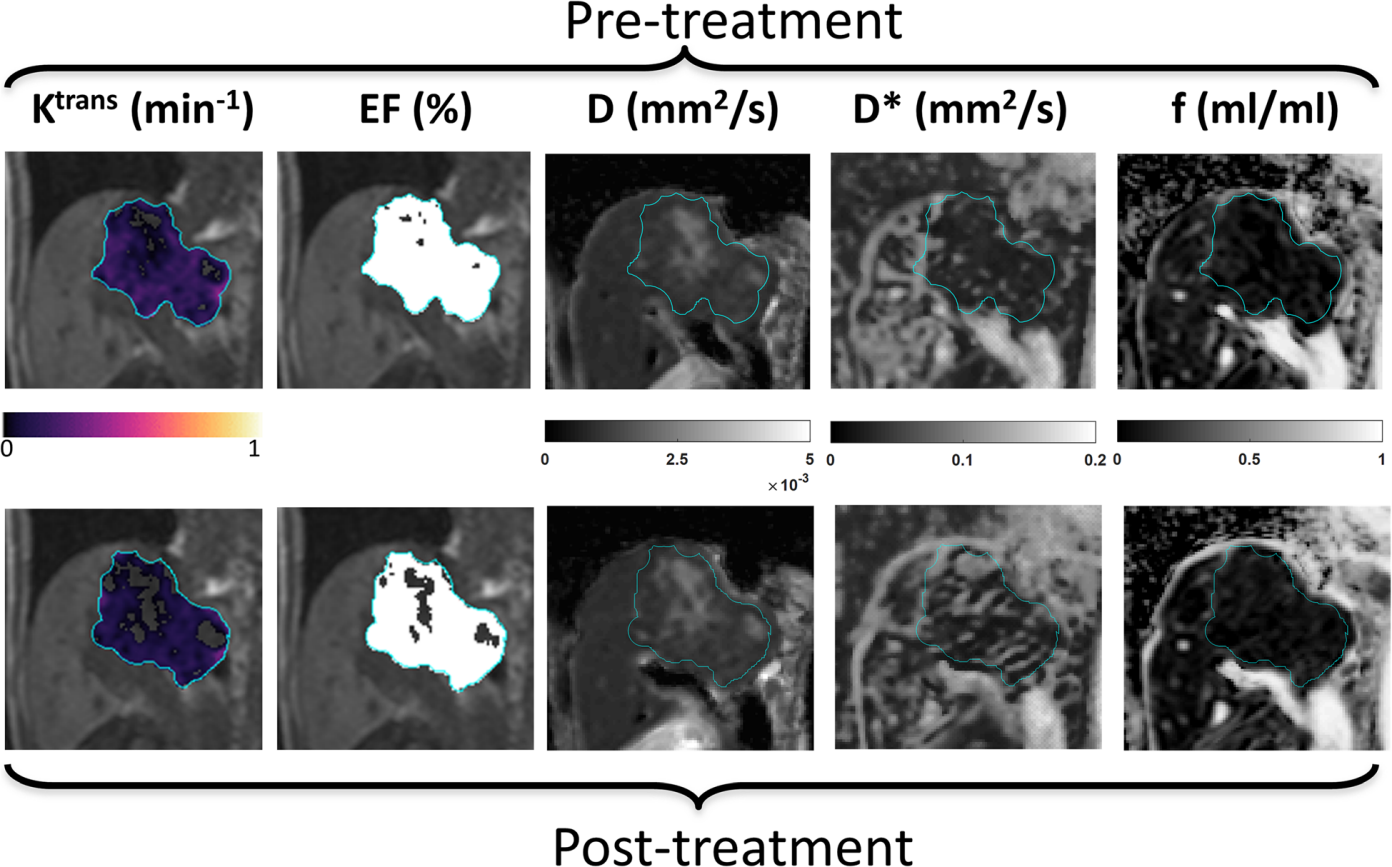
Example MR parametric maps from a 59 year old male patient with liver metastasis before (top row) and after 15 days of treatment (bottom row); coronal plane

The treatment response of the whole study cohort, as measured by DCE-MRI and IVIM-DWI, is shown in Fig. 3 using box plots overlaid with ladder plots for individual patients. Significant treatment-induced reductions of the perfusion-related DCE-MRI parameters were observed for EF (91.2% vs.50.8%, *P*<0.001), KEF (0.095 vs. 0.045, *P*<0.001) and K^trans^ (0.109 vs. 0.078 min^−1^, *P*=0.002). A typical example of MR parametric maps from a 59 year-old man with liver metastasis is shown in Fig. 4, demonstrating a decrease in K^trans^ and EF after treatment.

For IVIM-DWI, only the diffusion parameter, D, increased significantly post treatment (0.83 vs.0.97 × 10^−3^ mm^2^/s, *P*<0.001), in keeping with reducing cellularity. However, the perfusion-related parameter changes (f, D* and fD*) did not reach statistical significance. The detailed results including P-values of the pre/post-treatment Wilcoxon test of all MR parameters are presented in Table 3.

**Table 3:** Median values and main statistics of the DCE-MRI and IVIM-DWI parameters (Wilcoxon test; paired t-test and Bland-Altman analysis). Values with the # symbol were statistically significant at *P*<0.05.

Main cohort (*n*=25)	K^trans^ (nz)	EF	KEF	D	D*	f	fD*
Median value [Visit 1]	0.109	91.2	0.095	0.00083	0.0166	0.0947	0.0014
Median value [Visit 2]	0.078	50.8	0.045	0.00097	0.0095	0.1091	0.0011
P value (Wilcoxon signed-rank test)	0.002^*#*^	<0.001^*#*^	<0.001^*#*^	<0.001^*#*^	0.14	0.43	0.09
**IVIM Repeatability cohort (*n*=5)**	**K**^**trans**^**(nz)**	**EF**	**KEF**	**D**	D*	**f**	fD*
Mean value [Repeat 1]	x	x	x	0.0012	0.0348	0.0885	0.0027
Mean value [Repeat 2]	x	x	x	0.0012	0.0404	0.1065	0.0045
P value (paired t-test)				0.57	0.61	0.44	0.41
*Bland-Altman tests*							
standard deviation (SD)				0.00003	0.022	0.047	0.004
mean bias				-0.00001	0.006	0.018	0.002
coefficient of variation (CV) %; SD/mean				2.9	60	48	117

*nz=nonzeros*

### Correlation of DCE-MRI and IVIM-DWI parameters

**Fig. 5:**
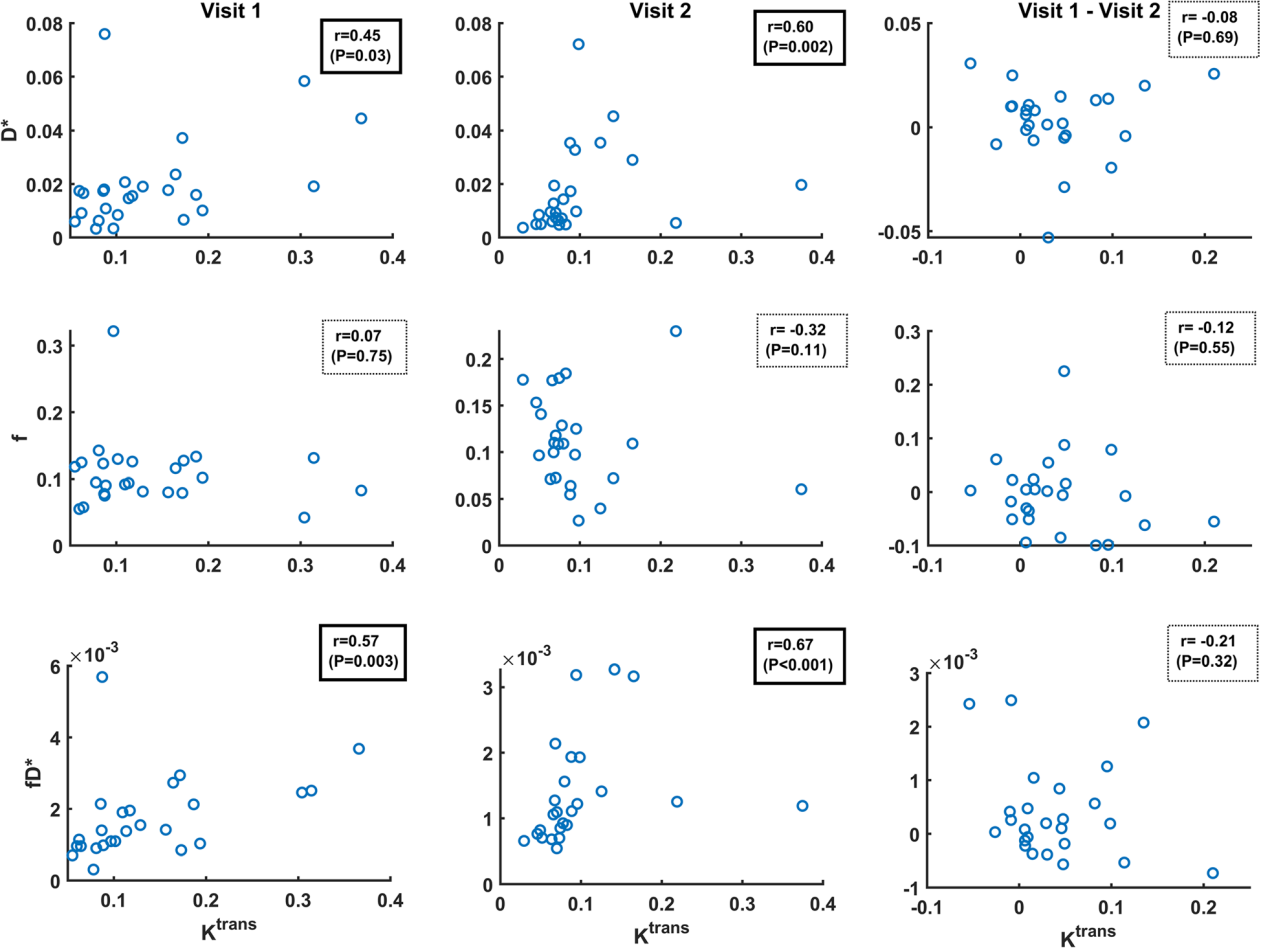
Scatter plots for IVIM-DWI perfusion parameters (D*, f and fD*) versus the DCE-MRI parameter (K^trans^) showing little correlation. Spearman’s rank correlation coefficient r and its corresponding P-value are shown for each plot. Only the four cases within a box framed with a solid line (i.e. D* and fD* versus K^trans^) were statistically significant. Pre- and post-treatment data from the main cohort of 25 patients with liver metastases

Scatter plots for the IVIM-DWI perfusion parameters (D*, f and fD*) versus the DCE-MRI parameter K^trans^ are presented in Fig. 5. Spearman correlation tests found no strong (i.e. correlation coefficient r>0.7) correlation between K^trans^ and IVIM-DWI parameters. A moderate correlation (0.5<r<0.7) was found, after treatment, between K^trans^ and two perfusion parameters: D* (*r*=0.60; *P*=0.002) and fD* (*r*=0.67; *P*<0.001). No significant correlation was found between f and K^trans^ at any timepoint during treatment.

### Repeatability of IVIM-DWI parameters

**Fig. 6:**
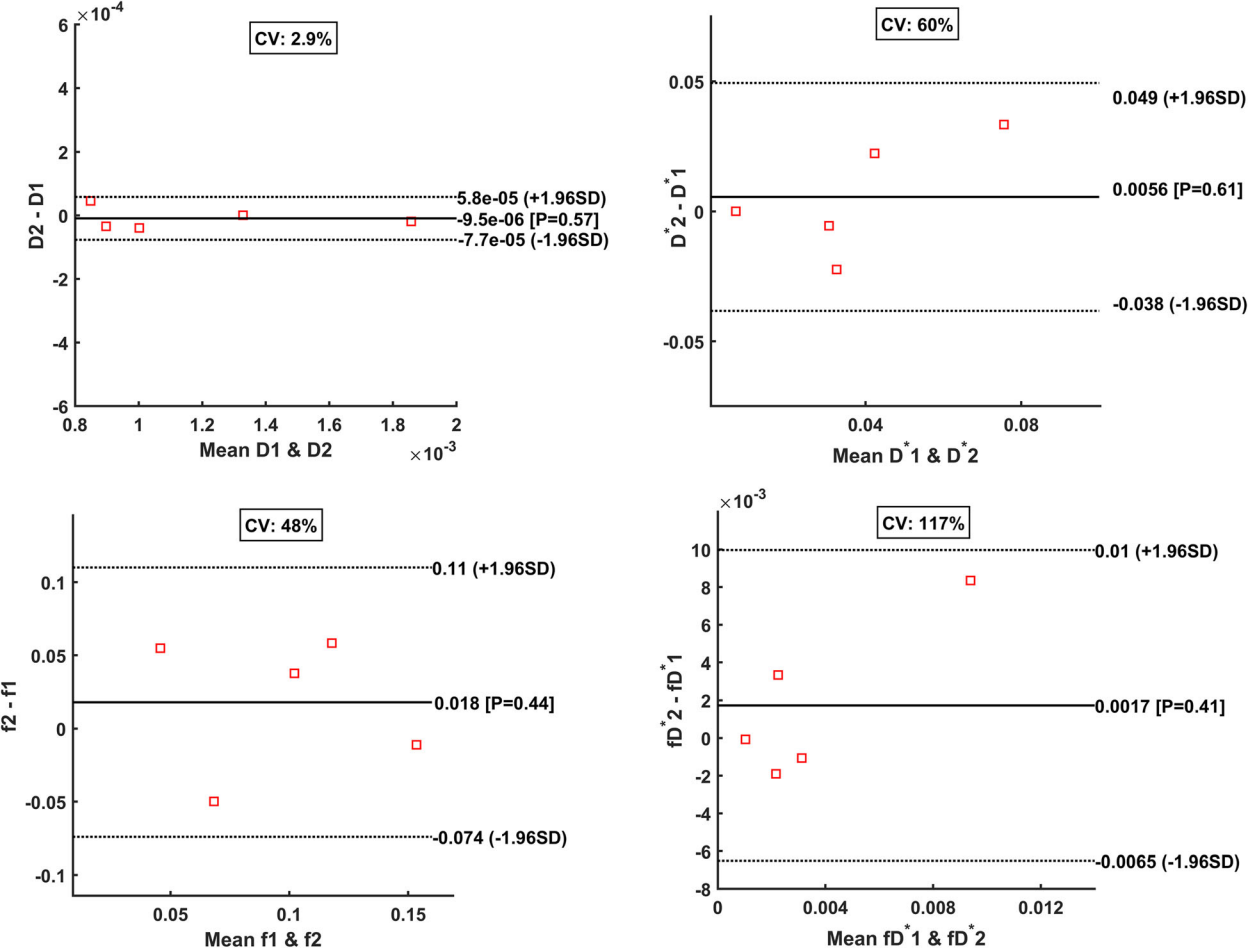
Bland-Altman plots for each IVIM-DWI parameter presenting values for the mean bias (and its P value), upper and lower limits of agreement and coefficient of variation. Data derived from the 5 patient repeatability cohort

Individual Bland Altman plots for each IVIM-DWI parameter as derived from the five patient cohort are presented in Fig. 6. The IVIM-DWI parameter repeatability was lowest for D (coefficient of variation CV=2.9%), followed by f (CV=48%), D* (CV=60%) and fD* (CV=117%). Full statistical results are presented in Table 3. The perfusion-sensitive parameters of colorectal liver metastases were considerably less repeatable compared with tissue diffusivity measurement (D).

### Correlation of MR parameters and RECIST

**Fig. 7:**
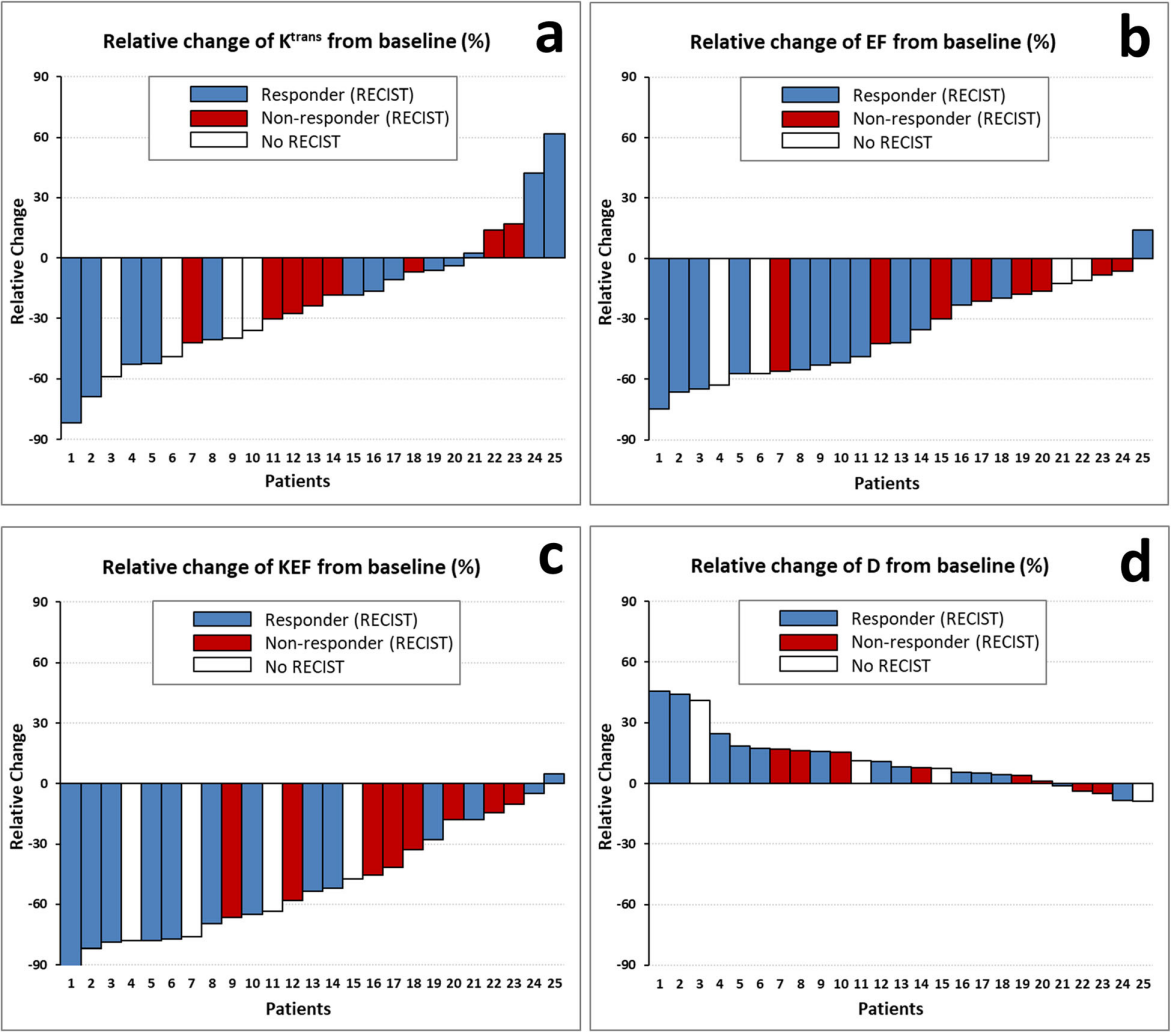
Waterfall plots for the four most sensitive MR parameters: K^trans^ (**a**), EF (**b**), KEF (**b**) and D (**d**) demonstrating a good identification of responders when using KEF and D parameters. The percentage change (relative to baseline value) for each MR parameter was calculated at day 15 post-treatment (25 patients), while the RECIST was performed at week 8 (21 patients)

Figure 7 presents waterfall plots of the four MR parameters showing statistical significance in assessing early tumour response at day 15 post-treatment: three DCE-MRI parameters (K^trans^, EF and KEF) and the non-perfusion D parameter derived from IVIM-DWI. Note that the disease control rate, measured by Response Evaluation Criteria In Solid Tumors v.1.1 (RECIST), was obtained at a later timepoint (week 8 post-treatment) and covered 21/25 patients as four patients were not evaluated by RECIST. Out of the 21 patients with RECIST v.1.1 evaluations available, KEF correctly identified 12/13 clinical responders and incorrectly identified 8/8 non-responders, while D correctly identified 11/13 responders and incorrectly identified 6/8 non-responders.

## Discussion

This prospective single-center study was performed in 25 patients with colorectal liver metastases treated with single-agent Regorafenib (anti-angiogenic and anti-proliferative effects). Across the cohort, there was a significant response measured by DCE-MRI demonstrating an overall decrease in the median K^trans^, EF and KEF values after treatment. This early decrease of DCE-MRI parameters was correlated, at later timepoints, with standard clinical biomarkers, including the endothelial marker CD31 which confirmed the anti-angiogenic effect of the drug in a smaller subcohort [6].

Additionally, the statistically significant functional MR parameters (K^trans^, EF, KEF and D) showed a good correlation with RECIST v.1.1 measurements (Fig. 7). Both KEF and D parameters correctly identified most clinical responders (12/13 and 11/13, respectively), whilst incorrectly classifying most non-responders as responders. Note that MR measurements were performed 2 weeks post treatment and the RECIST v.1.1 evaluation after 8 weeks. Such a discrepancy limits the interpretation of these comparisons since the lesion behaviour over time cannot be fully assessed (e.g. an early responder at 2 weeks might translate into a non-responder at a later timepoint).

In this study, we investigated whether perfusion-related IVIM-DWI effects mirror the perfusion effects measured using DCE-MRI in a cohort treated uniformly with a drug that has anti-angiogenic effects. To ensure optimum comparison, both DCE-MRI and IVIM-DWI data were motion corrected before method-specific analysis. Both DCE-MRI and IVIM-DWI data were acquired in the coronal plane (where we expect the principle source of motion that affects liver imaging to be superior-inferior in the direction of respiratory motion) and thus allowing our in-plane registration and motion correction to minimize the effects of motion; see further details in the Supplementary material.

Interestingly, the highly significant therapeutic effects observed for DCE-MRI parameters such as K^trans^, EF and KEF following Regorafenib treatment were not matched by changes in the perfusion-related IVIM-DWI parameters (f, D* or fD*; none reached significance). The diffusion coefficient D, a non-perfusion IVIM-DWI parameter, significantly increased by 17% post treatment (*P*<0.001), suggesting a reduction in cellularity after treatment. This increase in D was within the capability of our measurement repeatability to confidently detect it. Overall, these results suggest that a multiparametric analysis based on the most sensitive MR parameters (the 3 DCE-MRI parameters and the D coefficient) might yield an improved performance in assessing response to anti-angiogenic treatment.

The highest correlation between DCE-MRI and IVIM-DWI methods was moderate (*r*=0.67, *P*<0.001) and correlated K^trans^ and fD* parameters, even though changes in fD* failed to reach statistical significance when measured before and after treatment. The overall results suggest that even though fD* may be related to K^trans^ in these patients, the DCE-MRI measurement is more sensitive than IVIM-DWI for assessing treatment-induced changes in tumour perfusion in colorectal liver metastases. This is further influenced by the fact that perfusion sensitive IVIM-DWI parameters have relatively poor measurement repeatability (i.e. coefficient of variance for fD* was 117%). Such values were consistent with data derived from larger cohorts reported in the past [13, 24]. This suggests that although IVIM-DWI has been applied for assessment across the body, its role in assessing the vascular properties of hypovascular disease may be limited due to the combination of diminished sensitivity and poor measurement repeatability.

Another factor to consider when applying IVIM-DWI is that the technique does not measure perfusion in the classical sense [25], even though a link between IVIM-DWI parameters and conventional MR perfusion has been hypothesized [26]. However, a few studies in the liver found no correlation between DCE-MRI and IVIM-DWI parameters in liver cirrhosis [27] or hepatocellular carcinoma [28]. In another recent study, only a weak correlation was observed between IVIM-DWI and enhancement ratio in hepatocellular carcinoma; nevertheless, DCE-MRI outperformed IVIM-DWI parameters for the identification of necrosis [29].

In terms of clinical translation, DCE-MRI has already been used to demonstrate the pharmacodynamic effects of anti-angiogenic response of other drugs such as Bevacizumb [23, 30] and Sorafenib [31], while the use of IVIM-DWI remains limited to exploratory research. Moreover, recommendations towards standardization of DCE-MRI in the liver have been published recently [32], suggesting a wider acceptance of the technique. A similar standardization for IVIM-DWI is not currently available, although [32] includes useful suggestions for DWI standardization as well.

There are a few limitations to our study. First, although we undertook a repeatability study for our IVIM-DWI protocol, we did not perform this for our DCE-MRI protocol. The patients in this study were already subjected to a significant number of interventions, and it was difficult to justify undertaking a repeat baseline DCE-MRI study requiring an additional injection of gadolinium contrast. Nevertheless, the liver DCE-MRI protocol was the same as in a previous DCE-MRI study (coronal liver acquisition; 13 patients; population-based arterial input function), where the test-retest repeatability of DCE-MRI was characterised. In that study, a CV of 7.5% was found for K^trans^ [33], which corresponds to limits of agreement of -19%, +23%, and we expect similar repeatability values in this study. The change in median K^trans^ in our study cohort was 28%, and the individual change in K^trans^ in 15/25 (i.e. 60%) patients was in excess of the previously reported limits of agreement, suggesting a true measured effect.

Second, this was a study in a relatively small population from a single centre. Multi-centre DCE-MRI studies are challenging to perform as there are significant technical and operational challenges in undertaking such studies. Nonetheless, with evolving techniques in acquiring high-temporal resolution and high-spatial resolution images of the liver in free-breathing, it is likely that future DCE-MRI quantification may use semi-automatic or automatic pipelines to allow DCE-MRI parameters to be more widely evaluated in a multi-centre setting. Having said that, in the context of a larger cohort, the perfusion-related IVIM-DWI parameters could reach statistical significance in assessing therapy response.

## Conclusions

In conclusion, in our study of patients with colorectal liver metastases, IVIM-DWI perfusion-related parameters show limited sensitivity to the anti-angiogenic effects of Regorafenib treatment and showed low correlation with DCE-MRI parameters, despite profound and significant post-treatment reductions in DCE-MRI measurements. As such, IVIM-DWI parameters are not currently recommended as an alternative to contrast-based studies when assessing the changes in the vascular properties of colorectal liver metastases in response to treatment with an anti-angiogenic agent.

## Supplementary information

**Additional file 1**

## Data Availability

The datasets generated and/or analysed during the current study are not publicly available as appropriate regulatory and institutional approvals are required; datasets might be available from the corresponding author on reasonable request.

## Abbreviations

DCE-MRI: Dynamic Contrast Enhanced - MRI
IVIM-DWI: Intravoxel Incoherent Motion - Diffusion Weighted Imaging
D: diffusion coefficient
f: perfusion fraction
D*: pseudodiffusion coefficient
fD*: product of perfusion fraction and pseudodiffusion coefficient
K^trans^: volume transfer constant between plasma and extracellular extravascular space
EF: enhancement fraction
KEF: product of K^trans^ and EF
WHO: world health organisation
VIBE: volumetric interpolated breath-hold examination
VOI: volume of interest
R: Spearman correlation coefficient
CV: coefficient of variation
RECIST: Response Evaluation Criteria In Solid Tumors
