# Supplementary material for: DCE-MRI is more sensitive than IVIM-DWI for assessing anti-angiogenic treatment-induced changes in colorectal liver metastases
Source: Cancer Imaging. 2021 Dec 19;21:67. doi: 10.1186/s40644-021-00436-0 (PMC8684660; doi:10.1186/s40644-021-00436-0)
Supplement: Supplementary file 1 — Additional file 1 [file 40644_2021_436_MOESM1_ESM.docx]

**Supplementary Material**

**1. Motion Correction of MR data**

**In the liver, the dominant source of motion is from breathing, and the bulk of this motion is along the head-foot axis. The DCE-MRI and IVIM-DWI scans were both acquired coronally, so that the lesion displacements between time-points or b-values are largely in plane.**

**1.1 DCE-MRI**

**The DCE-MRI scans were acquired under sequential breath-hold at expiration, which minimises intra-frame motion artefacts at source, and ensures that all time-points are acquired in the same phase of the breathing cycle. Nevertheless, small changes in the lesion location remain, and these are almost all within +/-2 pixels of a suitably chosen reference frame. This residual motion was accounted for by manually shifting each target volume to best match the reference volume at the location of the lesion. The motion correction was restricted to whole-pixel shifts along the rows and columns, so that image interpolation was not required. Applying this process typically took less than five minutes per scan using in-house software written in MATLAB.**

**1.2 IVIM-DWI**

**The IVIM-DWI scans were acquired under free breathing, which results in larger displacements than for the DCE-MRI, but, with coronal imaging, the motion is still largely in plane. Due to the large number of images per scan needing registration (2200 = 20 slices × 5 repeat averages × 22 diffusion encoding gradients: b=0, plus seven non-zero b-values acquired in three orthogonal directions), a manual approach was unfeasible, so an automatic procedure was developed.**

**Motion correction was initiated by manually placing a rectangle over the lesion, ensuring that it encompassed the lesion on all slices. This box was then expanded by 25 pixels, and the computation of the registration cost functions described below were restricted to pixels inside the box. For each registration step, the optimal displacement was determined by exhaustive search of whole pixel shifts in the range +/-20 pixels (head-foot) and +/-10 pixels (right-left).**

**The following procedure was applied for each slice.**

1. **For each b-value group:**
   1. **Each image was registered to the b-value group median image using sum of absolute differences (SAD) cost function.**
   2. **The image with the lowest cost was chosen as the target image for the group.**
   3. **All images in the b-value group were registered to this target image using SAD cost function.**
   4. **The median image of the registered images was computed for the b-value group.**
2. **The b=240 s/mm^2^ median image computed in step 1d was used as the target image, to which the median images for the remaining b-values computed in step 1d were registered, using a mutual information cost function.**
3. **All source (unregistered) images were registered to the image with matching b-value resulting from step 2 using a SAD cost function.**

**2. Markov Random Field Model for IVIM Estimation**

**IVIM model fitting has been shown to give improved estimates by using spatial regularisation (1). This is achieved by augmenting the cost function in each voxel with a regularisation term that depends on the IVIM parameters values of the neighbouring voxels, and in this way spatial coherency of the IVIM parameter estimates is improved. We used a method very similar to the iterated conditional modes approach described in (2), but with a few modifications.**

**Firstly, we only applied the spatial regularisation to D* because we found that this was sufficient to stabilise the parameter fitting. D* is the rate of decay of the rapid component of the attenuation curve, and the fact that this is only captured by the first few b-values explains why unregularized estimates of D* have high errors. Once the estimates of D* are stabilised, the measured attenuation curve is sufficient on its own to give spatially stable estimates of D and f, and not applying spatial regularisation to these parameters avoids any additional bias (1).**

**Secondly, we applied the spatial regularisation to D* using a 3D Markov random field model, where the neighbourhood of each voxel includes the four adjacent voxels in the same slice and also the two nearest voxels in the slices above and below. Similar to (3), we chose the regularisation parameters by analysing the distribution of squared differences between neighbouring estimates of D* obtained without spatial regularisation (using a standard least-squares cost function). To account for the non-isotropic voxel size this was done separately for in-plane voxel interactions and through-plane interactions.**

1. While PT. A comparative simulation study of bayesian fitting approaches to intravoxel incoherent motion modeling in diffusion-weighted MRI. *Magn Reson Med*. 2017; 78(6):2373-2387. doi: 10.1002/mrm.26598. Epub 2017 Mar 31. PMID: 28370232.

2. Freiman M, Perez-Rossello JM, Callahan MJ, et al. Reliable estimation of incoherent motion parametric maps from diffusion-weighted MRI using fusion bootstrap moves. *Med Image Anal*. 2013; 17(3):325-336. doi:10.1016/j.media.2012.12.001

3. Kelm, B.M. et al. “Estimating Kinetic Parameter Maps From Dynamic Contrast-Enhanced MRI Using Spatial Prior Knowledge.” *Medical Imaging*, IEEE Transactions on 28.10 (2009): 1534-1547.
